# Investigations of the Tribological Performance of A390 Alloy Hybrid Aluminum Matrix Composite

**DOI:** 10.3390/ma11122524

**Published:** 2018-12-12

**Authors:** Abhilash Edacherian, Ali Algahtani, Vineet Tirth

**Affiliations:** 1 Mechanical Engineering Department, College of Engineering, King Khalid University, Abha 61413 Asir, Saudi Arabia; vtirth@kku.edu.sa; 2 Research Center for Advanced Materials Science (RCAMS), PO Box 9004, King Khalid University, Abha 61413 Asir, Saudi Arabia; alialgahtani@kku.edu.sa

**Keywords:** aluminum matrix composites, hybrid, A390, SiC, Gr, MoS_2_, tribological properties

## Abstract

Several challenges stand in the way of the production of metal matrix composites (MMCs) such as higher processing temperatures, particulate mixing, particulate–matrix interface bonding issues, and the ability to process into desired geometrical shapes. Although there are many studies showing composites with single particulate reinforcements, studies on composites with multiple reinforcing agents (hybrid composites) are found to be limited. Development of a hybrid particulate composite with optimized mechanical and tribological properties is very significant to suit modern engineering applications. In this study, Al–Si hypereutectic alloy (A390) was used as the matrix and silicon carbide (SiC), graphite (Gr), and molybdenum di-sulphide (MoS_2_) were used as particulates. Particulate volume (wt %) was varied and sample test castings were made using a squeeze casting process through a stir casting processing route. The evaluation of the mechanical testing indicates that the presence of both the hard phase (SiC) and the soft phase had distinct effect on the properties of the hybrid aluminum matrix composites (HAMCs). Composite samples were characterized to understand the performance and to meet the tribological applications. The 3D profilometry of the fractured surfaces revealed poor ductility and scanning electron microscopy fractography study indicated an intra-granular brittle fracture for HAMCs. Also, the dry sliding wear tests indicated that the newly developed HAMCs had better tribological performance compared to that of A390 alloy.

## 1. Introduction

Aluminum matrix composites (AMCs) have received great attention in the market of modern engineering materials which mainly focus on cost, quality and durability, styling and performance, emission and fuel economy, and recyclability [1]. Aluminum matrix composites with appropriate reinforcements offers several advantages such as high specific strength, hardness, stiffness, high-thermal and electrical conductivity, low coefficient of thermal expansion, good corrosion and wear resistance, etc. [2]. The reinforcement in AMCs may be monofilaments, continuous or discontinuous fibers, short fibers or whiskers, and fiber preforms and particulates [3,4]. A literature review indicates the dominance of particulate reinforced AMCs due to their ease of manufacturing and process competitiveness. This type of composite is produced by (i) solid-state processing such as powder metallurgy techniques, physical vapor deposition, diffusion bonding, etc. [5]; (ii) liquid-state processing such as stir casting, squeeze casting, melt-infiltration, spray deposition, etc. [6]; and (iii) in situ processing such as directional solidification of eutectics and formation of intermetallic phases [7]. Hard ceramic particulates such as carbides, oxides, nitrides, and borides have been widely used as reinforcements in AMCs. Generally used reinforcement constituents in aluminum alloy matrices are non-metallic phases such as SiC, Al_2_O_3_, MgO, TiC, TiB_2_, B_4_C, BN, AlN, or dissimilar metallic phases (Pb, Be, Mo, Ti, W, etc.) [8,9]. The addition of these particulates have been found to improve the properties of commonly used matrix alloys of series 2xxx, 5xxx, 6xxx and 7xxx (wrought alloys) through various strengthening mechanisms. However, to improve the tribological performance of the AMCs, researchers have used soft-phase solid lubricating agents such as graphite and MoS_2_ [10,11,12]. A few proven applications of AMCs include pistons, connecting rods, brake drums and rotors, braking systems of trains and cars, and recreational products such as golf club shafts and heads, ice-skating shoes, baseball shafts, horseshoes, bicycle frames, etc. [13,14,15]. Also, particulate reinforced composites have been successfully used in fan exit guide vanes in gas turbine engines as ventral fins and fuel access cover doors, flight control hydraulic manifolds in military aircrafts, and as rotating blade sleeves in helicopters [16,17,18]. Growing engineering and technology needs demand newer materials of superior performance. Hybrid composite materials, another class of composite materials which uses multiple reinforcements, are promising solutions for future material requirements to replace heavier materials and which suit a wide range of engineering components. However, making these components an exact shape and size from bulk composite materials has been a challenge over the years. Other challenges in the development of hybrid aluminum matrix composites (HAMCs) include inferior ductility, low fracture toughness, and precise control of the distribution of the different micro-constituents during processing. Many of the traditional metallurgical methods may be conveniently used to produce HAMCs. For example, the stir casting process may be followed by cost-effective casting methods such as gravity die casting (GDC), permanent mold casting (PMC), or squeeze die casting (SDC) to obtain HAMC components. In the present work, HAMCs are developed using A390 alloy as the matrix and silicon carbide, graphite (Gr), and molybdenum di sulphide (MoS_2_) as the particulate constituents using a stir casting process followed by a squeeze casting technique.

Tribological products are finding significance in industries and provide means to conserve energy and materials. An understanding of various friction and wear properties is necessary to make the right selection of materials and operating conditions for a given engineering application. Friction is a serious cause of energy dissipation, and wear is the main cause of material wastage in any system with mating bodies. Appropriate selection of materials for mating bodies and solid/liquid lubrication may be used to control friction and wear to an acceptable level. Mostly, monolithic materials are either incapable of satisfying the desired design requirements or are too expensive to meet superior tribological performance whereas HAMCs may be designed to meet this desired performance.

This investigation is focused on the study of composites using A390 aluminum cast alloy, as this alloy has wide acceptance in tribological applications such as cylinder blocks, transmission pump and air compressor housings, small engine crankcases, and air conditioner pistons [19]. This work includes the designing of a composite materials system (by varying both hard and soft particulate constituents wt %) at a compatible processing condition. There is no doubt that for the development of an appropriate HAMC, a number of experiments which quantifies the various properties such as mechanical and wear properties are required. Characterization studies mainly include metallurgical analysis to understand different phases and measurements of important properties such as tensile, compressive, impact strength, hardness, and wear. Further, the experimental observations in the production and characterization of HAMCs is expected to serve as a qualitative and quantitative guide to understand the effect of particulates in rendering various properties.

## 2. Materials and Methods

Among the various alloys of Al, Mg, Cu, Ni, Ti, and Fe, the Al alloys are the most popular matrix for MMCs. Aluminum matrix composites offer a range of mechanical properties depending on the chemical composition of the Al alloy. Mostly, used matrix alloys are wrought alloys such as 2xxx or 6xxx or cast alloys such as 2xx (Al–Cu) and 3xx (Al–Si–Cu) systems. They are usually reinforced by cost-effective particulates such as SiC, Al_2_O_3_, and TiC. The work during the nineties has shed light on many aspects of aluminum alloys and has classified Al–Si alloys as wrought alloys (0–1.65% Si) and foundry alloys (5–18% Si). Considering A390 alloy as a “quasi-binary alloy” (with Si as a major alloy content), it belongs to a hyper-eutectic group of Al–Si alloy systems. As a hyper-eutectic foundry alloy, A390 alloy (designated as per American Society for Metals) is commercially important due to its high strength-to-weight ratio and wear resistance. Table 1 shows the chemical composition of A390 alloy used in this study.

The particulate reinforcements in HAMCs may be either hard ceramic particulates (oxides, carbides, nitrides, borides, and silicide of mostly refractive metals) or soft self-lubricating non-metals (MoS_2_, Gr, talc, and other salts). Both MoS_2_ and graphite have lubricating abilities because of their layered lattice structure. Table 2 shows a few properties of materials used in this investigation. Through adequate control of particulate distribution within the bulk of the components, tailored properties like wear resistance and solid lubrication may be rendered to HAMCs, preserving other relevant properties of the matrix alloy. Among the various liquid state processing of composites, HAMC components may be conveniently made by using liquid-metal stir casting followed by squeeze casting. In stir casting, the reinforcement particle phase is dispersed in the melt by following a vortex method (manual or ultrasonic) and is allowed to solidify in a metal mold under pressure (direct squeeze casting). As an economical and effective processing route, many researchers have used this method to produce HAMCs [10,11,12].

**Figure 1:**
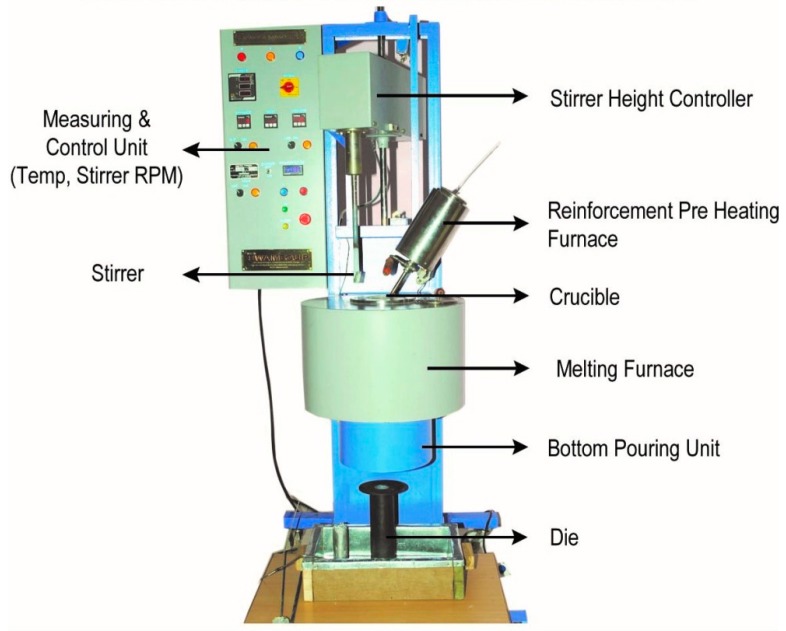
Stir casting setup used for manufacturing HAMCs (Courtesy: SwamEquip, Chennai, India).

In this investigation, a commercial aluminum (A390 alloy) ingot was melted using an induction furnace following standard melting practice [20]. In each experiment, desired quantity of preheated particulates (300 °C) were purged into the furnace and the molten alloy was stirred (at a rotation speed of 100 rpm) and poured under gravity at a temperature of 800 °C into the steel molds of cylindrical cavity to solidify under pressure (5–10 MPa) in order to obtain the test castings. Figure 1 shows the processing setup used for the present study. Two processing methods, gravity die casting (GDC) and squeeze die casting (SDC), were used to understand the influence of processing in the development of composites. To obtain squeeze cast samples, an additional setup was made for pouring the melt into the steel mold and forging and solidifying it under hydraulic pressure. In order to properly mix the preheated particulates, both motorized stirring and ultrasonic stirring were used. No refiner or modifier was added to the melt and the melt preparation was done with great attention to obtain defect free samples by avoiding slags and oxide layers.

The HAMC samples developed under this study followed the addition of particulates by varying its wt % according to the melt volume required for the mold. Two additional AMCs were developed to understand the effect of hard and soft phase on the properties of HAMCs. Table 3 shows various material samples designed in this work to effectively assess the properties of HAMCs. Higher percentages of the reinforcing agents in the matrix generally deteriorates the properties of the composites by forming oxide inclusions and porosity within the samples. Hence, a lower wt % of the constituents in the composite design was conceptualized in order to achieve the best performance of graphite and MoS_2_ in the presence of SiC [10,11].

Major characterization studies included the surface characterization (i.e., light microscopy, scanning electron microscopy (SEM), and 3D-profilometry) and evaluation of the mechanical and tribological properties. Procedures (i.e., sectioning, mounting, grinding, polishing, and etching) as per ASTM-E3 standards were followed to obtain vivid micrographs. Etching of aluminum alloys was usually done by immersing the polished surface in the etching reagent for 10–40 s. In this work, diluted hydrofluoric acid (HF (48%) + H_2_O) was used. After etching, every specimen was washed thoroughly with distilled water and then moped with pure cotton wetted with acetone to remove the water content and subsequently dried using a hot air blower. Microstructures of the etched samples were observed under an optical microscope and the photomicrographs were obtained using a high-resolution camera attachments. In addition to optical microscopy, wear samples were also examined with a high-resolution SEM (Hitachi SU6600, Tokyo, Japan) to obtain photomicrographs so as to analyze the wear surface. Scanning electron microscopy studies also included fractography to provide a comprehensive description on fracture mechanisms and morphologies on the tensile fractured specimens.

Each metallurgical sample prepared as per ASTM-E3 standards was subjected to Vickers hardness measurements following ASTM-E92 standards. The hardness test (with diamond indenter having angle between opposite faces, θ = 136°) was done at different locations of the composite specimens using a test load of 1 kgf and 10 s dwell time. The tensile and compression test properties (ultimate tensile strength and compressive strength) at room temperature were measured by performing tests at a constant strain rate of 1 mm/min using specimens prepared as per ASTM E8 standards on a computerized tensile testing machine (Instron, Norwood, MA, USA) of 150 kN capacity. The Charpy v-notch test is a standardized high strain-rate test which determines the amount of energy absorbed by a material during fracture. This absorbed energy is a measure of the toughness and acts as a tool to study temperature-dependent brittle-ductile transition. Charpy impact tests were carried out for all the samples on an Armstrong impact testing machine at a room temperature of 29 °C.

## 3. Dry Sliding Wear Testing

**Figure 2:**
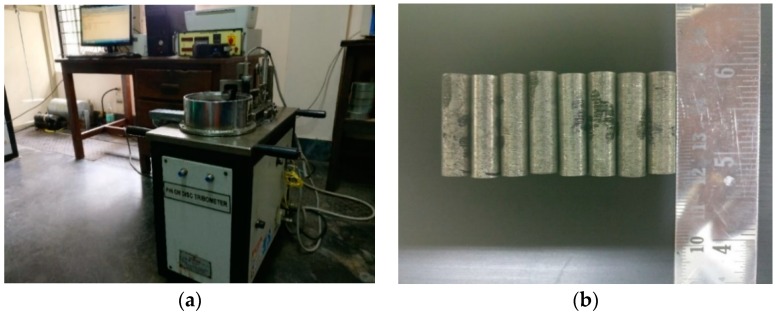
(**a**) Pin-on-disc tribometer used in this study and (**b**) a set of wear test specimens prepared as per ASTM G-99 standards.

**Figure 3:**
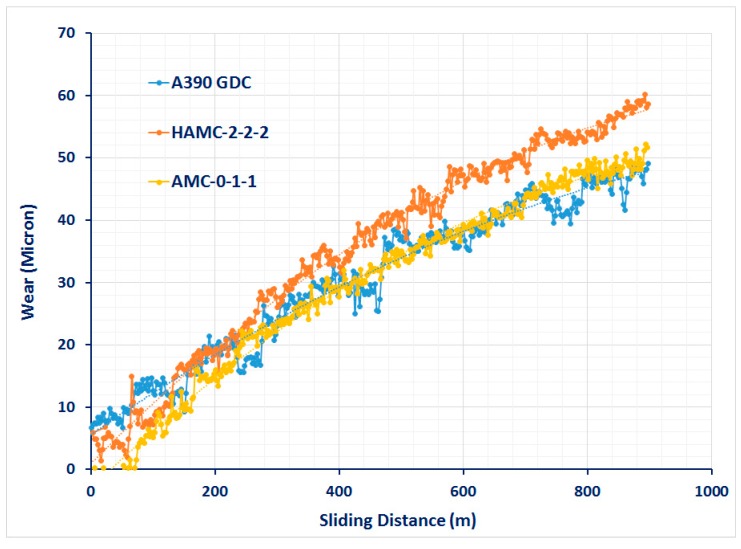
Wear behavior (computer generated) of a few test samples.

Tribological wear can be assessed either by ball-on disc type tribotesters [21,22] or by using pin-on-disc tribometers. In this study, cylindrical wear pins were prepared from three different sections of the composite castings to conduct wear test under dry sliding test conditions in compliance with ASTM G-99 standards using a pin-on-disc tribometer (shown in Figure 2a). Figure 2b shows a set of wear pins as per the standards (8 mm diameter and 27 mm length) made from each sample. Figure 3 shows a graph indicating the variation of height removed due to wear with respect to the sliding distance at a normal load of 10 N for a few as-cast samples. Since the computer-generated data was very sensitive, wear tests were conducted at various loads, i.e., 20 N, 30 N, and 40 N for a constant sliding distance of 1000 m and a constant sliding speed of 2.5 m/s to obtain wear properties based on the weight loss method. The selection of these testing parameters for the wear analysis was made based on erstwhile studies [11,12]. Before each experiment, the specimens were preheated and cooled to room temperature (28 °C) and the steel disc was ensured to be clean and dry. The track radius and time of the test was set according to the experiment and the K-type thermocouple was used to measure the temperature at 5 mm above the wear surface for each testing load just before the preset time elapsed. The wear volume loss was obtained from the displacement values given by the LVDT (linear variable differential transformer) attached with the tribometer. A SiC paper of 800 grid size was used to grind the face of each wear pin to match the average roughness (CLA 0.1 µm) of the rotating disc made of EN24 steel of hardness HRC 57.

## 4. Results and Discussion

In the initial experiments, there were a few defects, such as cold shut, due to decreased fluidity of melt added with SiC particles and raining due to improper melt pouring. Melt biting due to the oxidation of the constituents were also noticed. Ultrasonic stirring was done to prevent agglomeration and ensure effective mixing of soft constituents. It was noticed that ultrasonic stirring (using waves of frequency 18 to 20 kHz) was limited to a lower melt volume and temperature. The stirring time depended on the capability of the sonotrode horn to withstand the melt temperature. However, with the effective use of this new stir casting setup, it was demonstrated that HAMCs and AMCs may conveniently be fabricated from SiC, Gr, MoS_2_, and aluminum alloys using this advantageous casting route. Further, squeeze casting (melt forging) is a potential manufacturing process for producing defect-free casting parts. With proper operating parameters, quality HAMCs and AMCs with good dimensional accuracy may be produced using this process through a stir casting processing route.

**Figure 4:**
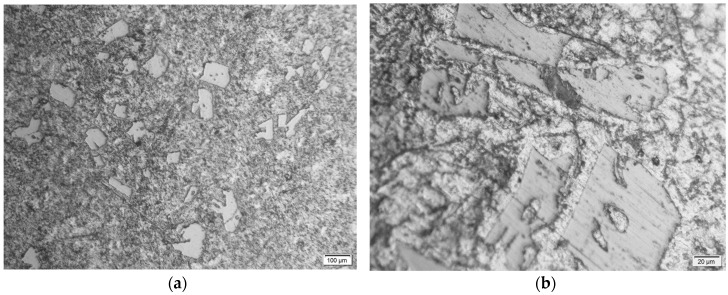
Photomicrographs (**a**) the distribution of particulates in the A390 alloy matrix (at 100X) and (**b**) the particulate morphology of SiC in the A390 alloy matrix (at 500X).

**Figure 5:**
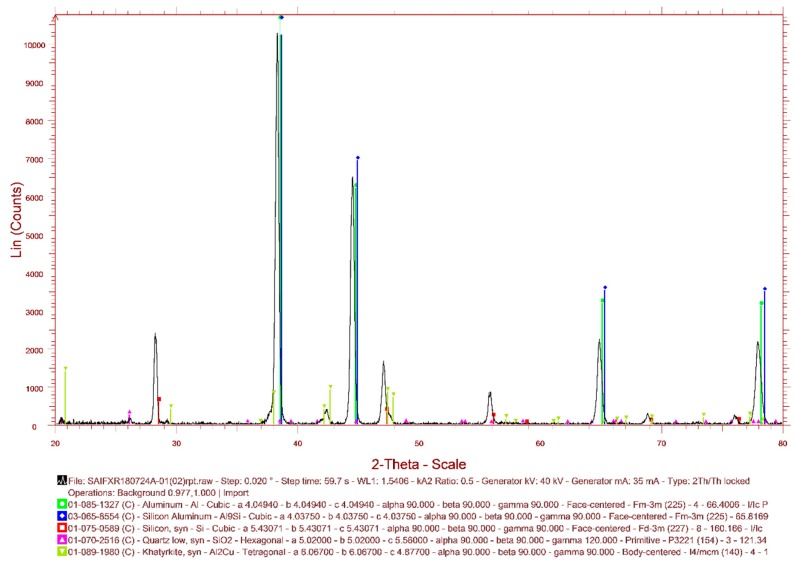
The X-ray Diffraction pattern of as-cast A390 cast sample.

From the metallurgical samples, very fine optical micrographs were obtained. Initially, the micrographs indicated an over etching using Keller’s reagent. This may be due to the presence of soft phase at the grain boundaries. However, the use of Dix–Keller’s reagent (2 ml hydrofluoric acid, 3 mL hydrochloric acid, 5 ml nitric acid, and 190 ml distilled water) helped to obtain a typical microstructure as shown in Figure 4. The micrograph of MMC with 2% SiC (220 mesh size) indicated banding of SiC (the white shiny particles) within the casting. A fine grading of faceted particulates was observed from the micrographs (see Figure 4a,b). The presence of black Gr and MoS_2_ can also be observed at the grain boundaries, which was not that distinct. Micrographs indicated the absence of porosity and an apparently uniform distribution of SiC in the A390 matrix. The absence of porosity within the HAMC samples indicate the efficiency of the squeeze casting process to manufacture defect-free composite products even without using any wetting agents. The X-ray Diffraction (XRD) pattern of as-cast A390 cast sample is shown in Figure 5. The XRD is not so sensitive to low phase contents; however, it indicated the presence of major elements Al and Si and the absence of intermetallic phases which may form during the processing of castings.

### 4.1. Hardness Test Studies

The average Vickers Hardness Number (VHN) values for a set of samples is shown in Table 4. It can be seen that hybrid AMC is harder than other samples. Also, it is noted that the hardness value increased with the increase in SiC particulate. However, only a little variation is noted in the AMCs having the presence of soft phase particulates Gr and MoS_2_. An average VHN value of 123 was observed for HAMCs, showing improvement over AMC with soft particulate constituent and A390 cast samples.

Hardness values obtained from Vickers hardness tests varied from 107–155 VHN. A variation of tensile strength values from 134 MPa to 160 MPa and hardness values VHN 70 to VHN 138 for Al–Si (4 to 20 wt %) system was noted from previous studies to compare the respective values obtained from the experimental studies. Higher values of VHN for A390 alloy were observed in the case of the squeeze cast samples compared to that of the gravity die cast samples. This indicates the process capability of squeeze casting in producing defect-free HAMCs.

### 4.2. Impact Test Studies

Charpy impact tests were carried out for all material samples (as-cast A390 GDC and SDC samples, AMCs, and HAMCs) on an Armstrong impact testing machine at a room temperature of 29 °C. As indicated by Table 4, the as-cast A390 (both GDC and SDC) samples showed higher impact energy than the AMC and HAMC samples. This also indicated a decrease of impact strength with the increase of wt % SiC in the matrix. Only a little variation in impact energy was observed among the samples. However, it is noted that the presence of soft phase particulates decreased the impact strength. The average impact energy of HAMCs was found to be 47.6 joules. This may be due to the ability of the particulates to propagate the crack. Impact strength of HAMCs was found to vary according to the wt % of the constituents and validates the rule of mixtures in the case of composites.

**Figure 6:**
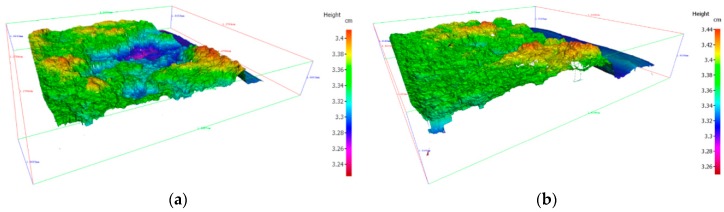
3D profilometry of Charpy test samples (**a**) A390-SDC, and (**b**) HAMC-4-1-1.

Also, the analysis of the fracture surface of the Charpy test samples revealed a mixed brittle-ductile fracture in the case of matrix alloy cast samples (indicating dull and little shiny dots with lots of dimples) and brittle fracture in case of HAMCs. Three-dimensional profilometry (Alicona) of these samples are shown in Figure 6. This indicates that the matrix alloy (A390 alloy) samples have more ductility compared to the HAMC samples.

### 4.3. Tensile and Compression Test Studies

**Figure 7:**
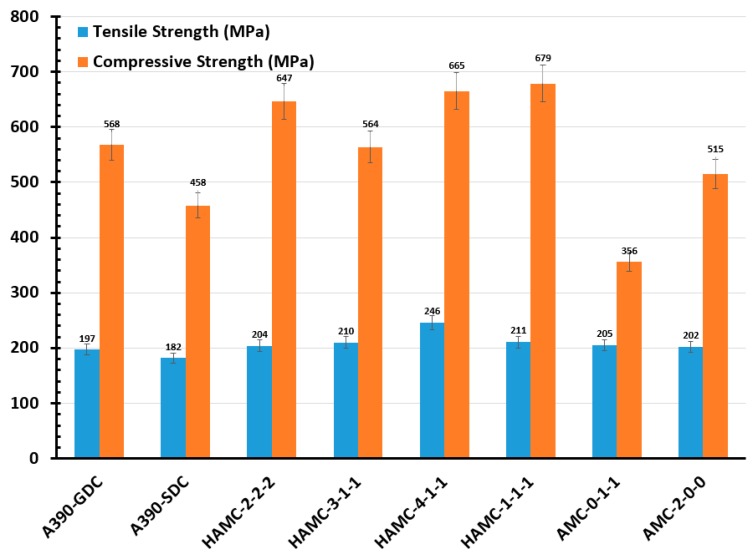
Average values of tensile and compressive strength of material samples.

According to the compositional variation introduced by the melt-treatment, the ultimate tensile strength (UTS) of material samples varied from 190 MPa to 250 MPa (see Figure 7). The microstructure containing small cells and Si particles developed damage at a lower rate, whilst the microstructure with large cell sizes and large or elongated Si flakes tended to crack at low strains, indicating a poor ductility. Accordingly, superior tensile test values were obtained for HAMC samples compared to other samples, especially as-cast A390 GDC and SDC samples, which may be due to the heterogeneous nucleation phenomena in composite samples. Similarly, UTS obtained from the compression test of the material samples varied from 450 MPa to 680 MPa (refer Figure 7). Least compressive strength was observed in the case of the sample AMC-0-1-1, indicating the incapability of soft phase to resist the compressive strength. Hybrid aluminum matrix composites showed better compressive strength compared to base alloy cast samples and AMC samples.

### 4.4. Dry Sliding Wear Studies

**Figure 8:**
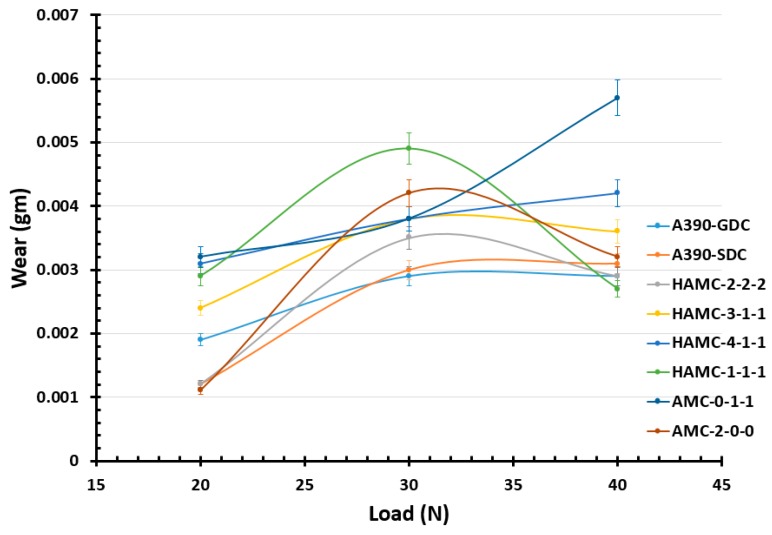
Plot showing the average wear loss of material samples at different wear loads.

The results obtained from computerized wear tests (wear height (µm) at various sliding distance (m)) were very sensitive to analyze and to understand the performance of each samples. Hence, to appropriately evaluate the wear property, a weight loss method was devised. It is evident from the Figure 8 that for all the cast samples, the wear volume increases as the normal load increases. Among all samples, the wear rate was found to be less for HAMCs for which the average weight loss was 0.0035 gm for a sliding distance of 1000 m at a sliding speed of 2.5 m/s.

The addition of reinforcement has been reported to enhance the wear properties of A390 alloys. The processing method, distribution of reinforcements, and reinforcement morphology is important in the determination of the wear mechanism. Wettability of particulates is an important parameter often discussed when dealing with particulate reinforced metal matrix composites. The better the wettability of particulates, the better the particulate matrix interface strength, which is a key factor in the effective transmission of load from the matrix to the particulate. In addition to the sliding conditions, the properties of reinforcements and matrix-like chemical affinity, work hardening ability, fracture toughness, and hardness also influences the wear mechanism of the composites. More analysis with XRD and SEM of wear debris and mechanically mixed layers (MML) are desirable to understand the composition and iron particle transfer to the pin surface contributing to the wear mechanisms of these materials. Table A1, Table A2 and Table A3 show the wear loss of material samples at a load of 20 N, 30 N, and 40 N, respectively, for a sliding distance of 1000 m at a sliding speed of 2.5 m/s.

The volume loss due to the wear of the samples was plotted against different loads for a constant sliding speed and distance, as shown in Figure 8. It is evident from Figure 8 that for all the materials up to a load of 30 N, the wear volume increases as the normal load increases. However, at higher loads, the volumetric wear loss of HAMCs is found to be less than that of matrix alloy (A390 aluminum alloy). Also, SiC reinforced AMC showed a lower wear rate than that of Gr & MoS_2_ reinforced AMC which showed a steeper wear volume loss as the test load increases. The volumetric wear loss of HAMCs is found to vary between that of AMC samples of soft and hard phase reinforcements. At a higher applied test load of 30 N and at constant sliding speed of 2.5 m/s, it can be considered that wear occurred in as-cast A390 samples is severe or plastically dominated and that in HAMCs and AMC is mild wear. However, these composites are susceptible to a two-body or three-body abrasive wear.

### 4.5. Fractography and Worn Surface Characterization

**Figure 9:**
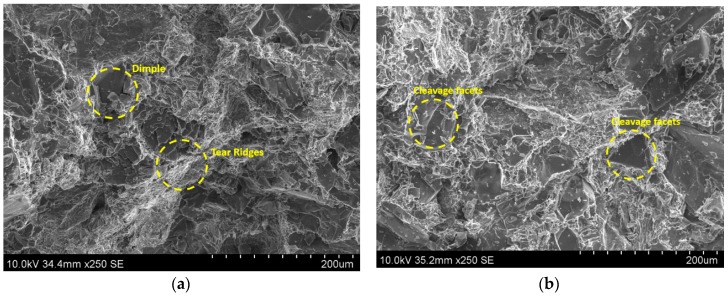
SEM photomicrographs of (**a**) gravity die cast A390 alloy showing many dimples and tear ridges and (**b**) squeeze die cast A390 alloy showing many cleavage surfaces and fewer tear ridges.

**Figure 10:**
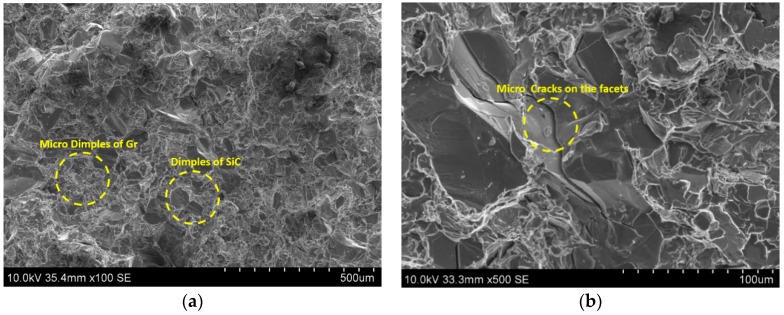
SEM photomicrographs of HAMCs (**a**) showing many dimples of particulates and fewer tear ridges and (**b**) cleavage facets and micro-cracks.

As understood from the literature the most likely factor causing fracture initiation in A390 alloy casting is the silicon particle cracking causing initiation of micro-cracks. The brittle nature of silicon needles and their sharp tips create zones of localized stress that are expected to contribute to deterioration of the tensile properties. The fracture of the A390 alloy was governed by the silicon phase itself. Usually, micro-cracks are initiated in the silicon phase and which then grow and link until the complete fracture occurs. A SEM photomicrograph of the gravity die cast A390 alloy showing many cleavage surfaces and tear ridges is shown Figure 9a. The presence of tear ridges (white lines) is an indication of higher deformation energy absorbed during the tensile fracture by the specimen. This fracto-micrograph also shows many regions of cleavage facets besides displaying small dimples of grains, and small microstructure discontinuities (gas pores or pores by particulates) showing a ductile-brittle nature of failure. The silicon phase forms a continuous network and the crack propagates on the silicon cleavage planes or other brittle microstructure components (intermetallic phase). The sharp edges of Si precipitates or ends of brittle SiC particles are preferred crack initiation sites generating cleavage facets. Figure 9a shows many dimples and cleavage facets due to Si flakes in GDC samples, whereas Figure 9b shows mainly the cleavage facets due to Si flakes in SDC samples. Figure 9b indicates lesser tear ridges compared to that in Figure 9a, indicating the poor ductility of squeeze cast samples. Many micro dimples due to the presence of Gr or MoS_2_ can also be seen in the Figure 10a. Similarly, the micro-cracks on the cleavage facets (Figure 10b) show the brittleness of the Si flakes in HAMCs. The visible secondary cracks and cleavage steps in the fracto-micrographs (Figure 9b and Figure 10a,b) confirm the trans-crystalline brittle fracture in both squeeze die casted A390 alloy and HAMCs. This also indicates that the decohesion mechanism and the crack paths are strongly influenced by the volume fraction and the morphology of particulates.

**Figure 11:**
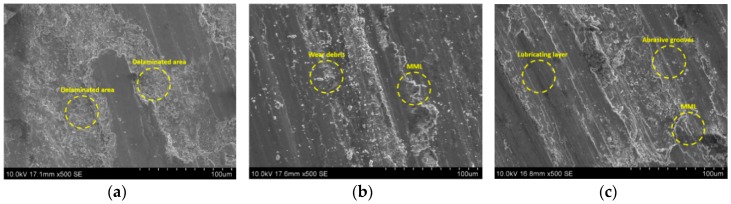
SEM photomicrographs of the worn surface of the sample (**a**) A390-GDC; (**b**) HAMC-4-1-1; and (**c**) HAMC-1-1-1.

The worn surface topography and the morphology of wear debris formed on it during pin-on-disc tests were studied using SEM. Figure 11a shows SEM photomicrographs of the worn surface of the A390-GDC sample at a normal load of 40 N. It shows the subsurface deformation of the sample showing micro-cracks and craters formed on the surface. On the other hand, the samples HAMC-4-1-1 (refer Figure 11b) and HAMC 1-1-1 (refer Figure 11c) indicate the formation of a mechanically mixed layer (MML) on the pin surface. The fine wear debris of soft particles reattached to the worn surfaces due to the Van der Waals force, as well as electrostatic force leading to the formation MML. The presence of MML in HAMC samples contributed to a lower wear rate at higher loads compared to that of other samples. The presence of a dark lubricating layer on the pin surface indicates the efficiency of Gr or MoS_2_ soft phase agents in reducing the wear in HAMCs which occurs mainly due to the abrasive grooves due to SiC debris. It can be inferred that the predominant wear mechanisms in HAMCs are brittle fracture due to two and three body abrasion of dislodged SiC, Gr, and MoS_2_ particulates, and the resulting fatigue and micro-cracking of MML.

## 5. Conclusions

Major conclusions from this study are:

- Defect-free hybrid metal matrix composites may conveniently be fabricated through stir casting processes followed by a squeeze casting technique. Micrographs showed uniform dispersion of the reinforcement particles SiC, Gr, and MoS_2_ in A390 alloy without any porosity.
- Hardness of the AMCs were found to be better than that of the A390 alloy, and was found to be increasing with the percentage of SiC, and the 3D profilometry of the fracture surface showed poor ductility for HAMCs.
- Presence of SiC in AMCs reduced the impact energy and it was further reduced with the addition of soft phase constituents like Gr and MoS_2_.
- Fractography of the tensile test specimens indicated intra-granular brittle fracture of AMCs and HAMCs.
- The wear study proposes that predominant wear mechanisms in HAMCs are brittle fracture due to two and three body abrasion of dislodged SiC, Gr, and MoS_2_ particulates, and the resulting fatigue and micro-cracking of MML. The HAMCs exhibited better dry sliding wear behavior than die cast A390 matrix alloy, indicating enhanced tribological characteristics of HAMCs with soft phase constituents such as Gr and MoS_2_ in the presence of hard phase reinforcements like SiC.

The study revealed the formation of dross and melt biting if the SiC addition was more, especially when it was added without purging. The limitation of ultrasonic stirring was assessed and this study suggests that it is feasible only for a small quantity of the melt at a lower range of operating temperature. Major recommendations include appropriate preheating and stirring to ensure proper mixing of particulate in the matrix and a corrosion study to understand the oxidation behavior of hybrid composite during tribological applications.

## Figures and Tables

**Table 1:** Chemical composition of A390 alloy.

Al	Si	Cu	Mg	Fe	Mn	Zn	Ti
75.3–79.5	16.0–18.0	4.0–5.0	0.50–0.65	0.40	0.10	0.20	-

**Table 2:** Properties of matrix alloy and constituent agents of hybrid aluminum matrix composites (HAMCs).

Property	Unit	A390	SiC	Graphite	MoS_2_
Density (at 20 °C)	g/cm^3^	2.73	3.22	2.09–2.23	5.06
Melting point	°C	650	2973	3915	1185
Coefficient of thermal expansion	µm/m∙°C	18	4	2–6	1–4
Thermal conductivity	W/mK	170	126	85	40
Young’s modulus	GPa	82	410	10	330

**Table 3:** Material samples developed for this investigation.

Sample No	Sample Code	SiC %	Gr %	MoS_2_ %	Process
1	A390-GDC	0	0	0	GDC ^#^
2	A390-SDC	0	0	0	SDC ^$^
3	HAMC-2-2-2	2	2	2	SDC
4	HAMC-3-1-1	3	1	1	SDC
5	HAMC-4-1-1	4	1	1	SDC
6	HAMC-1-1-1	1	1	1	SDC
7	AMC-0-1-1	0	1	1	SDC
8	AMC-2-0-0	2	0	0	SDC

^#^ Gravity Die Casting; ^$^ Squeeze Die casting.

**Table 4:** Average values of Vickers Hardness Number (VHN) and impact energy.

Sample Code	VHN Values	Impact Energy (N-m)
A390-GDC	107.3	49.03
A390-SDC	122.6	48.01
HAMC-2-2-2	117.8	47.07
HAMC-3-1-1	117.9	45.11
HAMC-4-1-1	127.9	52.95
HAMC-1-1-1	126.4	43.64
AMC-0-1-1	124.6	43.14
AMC-2-0-0	152.5	46.06

## Appendix A

**Table A1:** Wear loss of samples (Sliding Velocity: 2.5 m/s, Load: 20 N, Sliding Distance: 1000 m).

Sample No.	Initial Weight (gm)	Final Weight (gm)	Track Diameter (mm)	Test Speed (RPM)	Time (MM:SS)
1	3.7131	3.7112	60	795	06:40
2	3.6852	3.6840	70	682	06:40
3	3.5425	3.5413	80	596	06:40
4	3.6051	3.6027	90	530	06:40
5	3.7072	3.7041	100	497	06:40
6	3.8174	3.8145	110	434	06:40
7	3.6756	3.6724	120	397	06:40
8	3.6857	3.6846	90	530	06:40

**Table A2:** Wear loss of samples (Sliding Velocity: 2.5 m/s, Load: 30 N, Sliding Distance: 1000 m).

Sample No.	Initial Weight (gm)	Final Weight (gm)	Track Diameter (mm)	Test Speed (RPM)	Time (MM:SS)
1	3.6829	3.6800	60	795	06:40
2	3.6055	3.6025	70	682	06:40
3	3.5117	3.5082	80	596	06:40
4	3.5459	3.5421	90	530	06:40
5	3.5661	3.5623	100	497	06:40
6	3.7483	3.7434	110	434	06:40
7	3.5956	3.5918	120	397	06:40
8	3.6362	3.6320	90	530	06:40

**Table A3:** Wear loss of samples (Sliding Velocity: 2.5 m/s, Load: 40 N, Sliding Distance: 1000 m).

Sample No.	Initial Weight (gm)	Final Weight (gm)	Track Diameter (mm)	Test Speed (RPM)	Time (MM:SS)
1	3.6804	3.6775	60	795	06:40
2	3.6033	3.6002	70	682	06:40
3	3.5087	3.5058	80	596	06:40
4	3.5426	3.5390	90	530	06:40
5	3.5634	3.5592	100	497	06:40
6	3.7441	3.7414	110	434	06:40
7	3.5924	3.5867	120	397	06:40
8	3.6325	3.6293	90	530	06:40
